# Welding Temperature Distribution and Residual Stresses in Thick Welded Plates of SA738Gr.B Through Experimental Measurements and Finite Element Analysis

**DOI:** 10.3390/ma12152436

**Published:** 2019-07-31

**Authors:** Xiaoyu Yang, Guizhen Yan, Yanfei Xiu, Zhongwei Yang, Gang Wang, Wei Liu, Shaohua Li, Wenchun Jiang

**Affiliations:** 1 Shandong nuclear power equipment manufacturing co., LTD, Shandong, Haiyang 265118, China; 2 College of Mechanical and Electronic Engineering, China University of Petroleum (East China), Qingdao 266555, China; 3 School of Material Science and Engineering, North University of China, Taiyuan 030051, China

**Keywords:** SA738GR.B, 2-D model, heat source, residual stresses, thick welded plates

## Abstract

A numerical and experimental study of welding temperature distribution and residual stresses in thick welded plates of SA738Gr.B was conducted. Within the framework of numerical investigations, the temperature field of SA738 thick plate welding was simulated and analysed by using 2-D modelling technology. The temperature field was checked by using the thermal cycle curve with the aim of increasing the computational accuracy and efficiency, and the temperature field was verified by using the thermal cycle curve and heat affected zone. The welding stress field was analysed based on the temperature field, and the indentation test method was used to verify the stress field, and the error was controlled to within 12.5%. With the help of a welding model established for SA738Gr.B thick-plate welding the sequence was simulated. Seen from welding sequence 1 to welding sequence 3, transverse stress S11 changed significantly, decreasing by 14% and 17% respectively, adjusting the welding sequence can reduce welding residual stresses.

## 1. Introduction

Thick plates are widely used in nuclear power plants, ships, large bridges, etc. [1,2]. Uneven welding temperature distributions, and significant constraint conditions [3] in the thick-plate welding process mean that complex residual stresses are generated owing to the need for multiple welding passes. Numerical simulation has become an important tool to predict welding residual stress, but the ability to simulate the structure is always limited by available computing power in such transient non-linear models aimed at reducing the welding process, and time, complexity [4,5]. Many researchers also put forward some calculation methods to improve the accuracy of the calculation [6,7,8].

Theoretically, the numerical simulation analysis of welding is generally divided into two parts: the welding temperature field and the welding stress field [9,10,11,12,13]. The analysis of the temperature field is a representative problem concerning non-linear transient effects. During the movement of the heat source, the temperature of the whole welding piece changes with time and position, and the thermo-physical properties of the material also change with temperature. Meanwhile, latent heat and melting are also involved in this process. Therefore, the welding temperature field is both uneven and unstable [14].

Generally speaking, 3-D numerical models can accurately predict post-welding deformation and residual stress distribution [15,16,17,18,19]. Nevertheless, some studies have found that the results obtained by a 2-D model can be effective [20,21]. In addition, the duration of 3-D numerical simulations of the welding process remains a challenge, so a 2-D model is selected here.

Previous workers have carried out numerical simulation analysis of thick-plate welding of low-alloy high-strength steel, but did not establish the numerical model of the temperature field and stress field for thick-plate welding of SA738Gr.B. In this study, the physico-mechanical properties of SA738Gr.B material at different temperatures were tested, and the welding temperature field and stress field were established, and the welding sequence discussed.

## 2. Experimental Procedure

### 2.1. Welding Process

**Figure 1:**
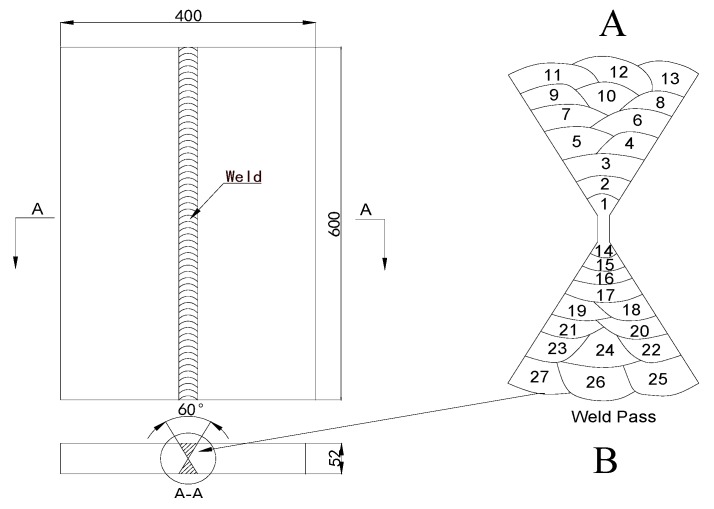
Dimensions of the 52-mm-thick plate.

Manual shielded arc welding (SMAW) was carried out on SA738GR.B [22] steel plates. The chemical composition of the base metal is summarised in Table 1. Welding was carried out using a matching welding material (E9018-G-H4, composition as given in Table 1; electrode diameter, 4.0 mm). The geometry of two plates when welded into an X-groove, including the relevant dimensions, are presented in Figure 1. The length, width, and thickness are 600, 400, and 52 mm, respectively. The welding procedure is listed in Table 2. A minimum preheat temperature of 100 °C and maximum inter-pass temperature of 200 °C were used.

### 2.2. Welding Temperature Field Measurement

**Figure 2:**
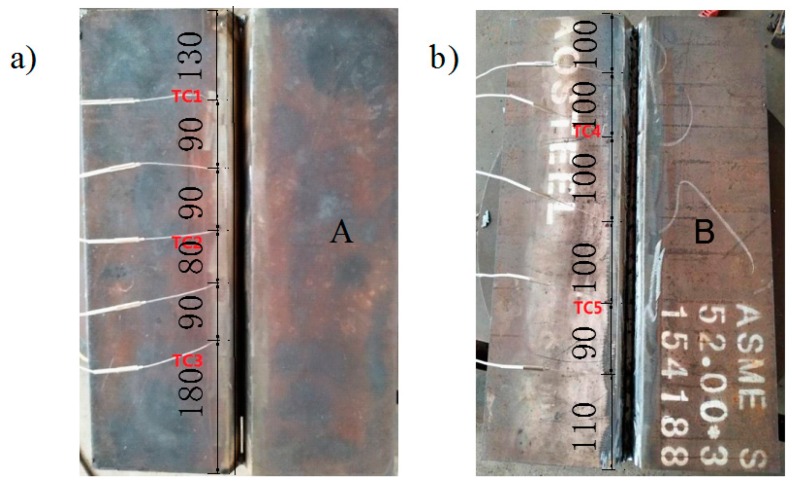
Distribution of temperature testing points: sides A (**a**) and B (**b**).

The temperature control detector was used to measure the temperature of welding plates. There are 10 terminals which can measure five points at the same time. As shown in Figure 2, three testing points (TC1 to TC3) were distributed along the seam line of side A and two testing points (TC4 and TC5) were distributed along the seam line of side B. The distance between measuring points TC1, TC2, and TC3 on side A, TC3 and TC4 on side B, and the centre of the weld line is 10 mm, that is, the distance between the three measuring points and the border of the weld line is no more than 5 mm. The longitudinal distance of thermocouple is indicated in Figure 2 (dimensions: mm). There were 10 thermocouples used in the temperature collection process, but only five actually worked (two were accidentally knocked off during welding, and three fell off when moving the test plate, leading to their failure to collect data).

### 2.3. Residual Stress Measurement

**Figure 3:**
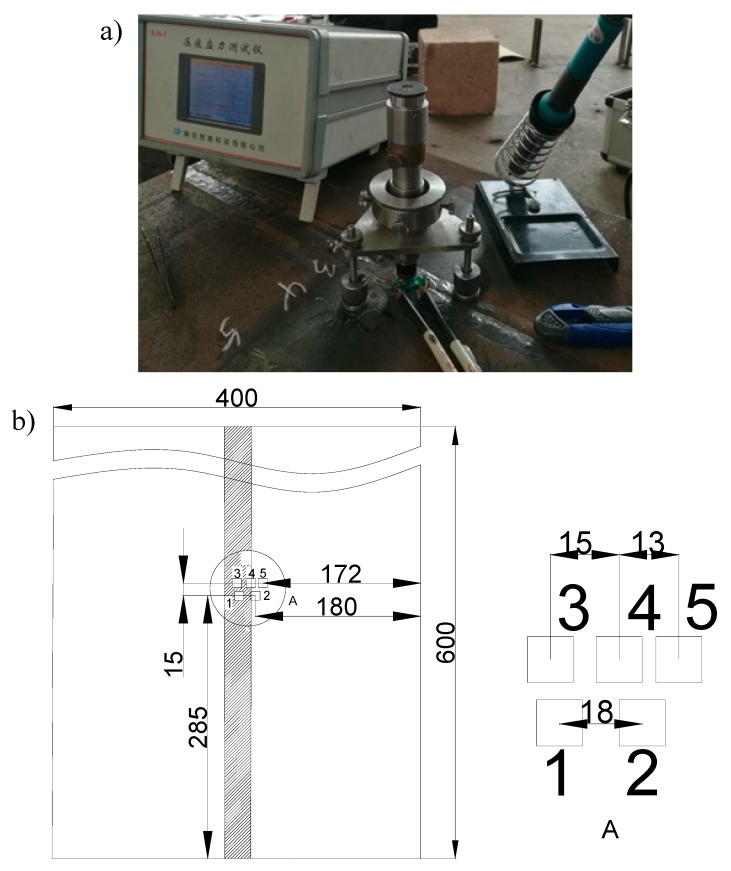
The experimental set-up (**a**) and distribution of measurement points (**b**).

The residual stress was measured according to the indentation method [23]. As shown in Figure 3, five testing points (1 to 5) were distributed along the seam and heat-affected zone.

## 3. FE Modelling

### 3.1. FE Mesh

**Figure 4:**
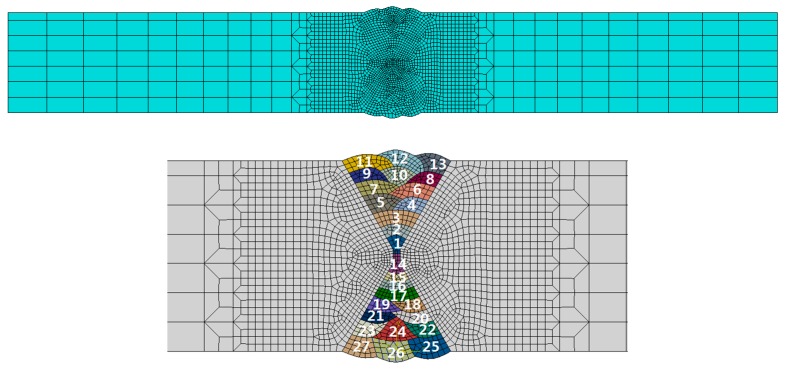
Grid diagram of welding model of the manual welding process.

In this study, multi-layer and multi-pass welding were used. When modelling the multi-pass welding process, the filler metal should be fully considered when calculating the height and volume of each pass. According to the actual weld section shape, the calculation models for manual shielded arc welding were established (Figure 4).

A 2-D FE (finite element) model was established in ABAQUS software: due to the high flux and temperature gradient in the fusion zone (FZ) and its adjacent regions, namely the heat-affected zone (HAZ), a finer mesh was used (the element size increases with distance from the welding centreline).

Welding simulation involved a sequentially coupled thermal-elastic-plastic behaviour model, where the thermal analysis was first completed and then the acquired temperature data were read into the mechanical analysis as an applied thermal load. The FE mesh and element birth and death used in the structural analysis are the same as that used in the thermal analysis. Nonetheless, both analyses have boundary conditions. The element types and dissimilar element types for thermal and mechanical analysis are CPE4R and DC2D4, respectively. In this FE model, the total number of elements was 2372.

### 3.2. Thermal Analysis

The maximum value of welding residual stress usually occurs in the middle of the sample, and the distribution size is similar. In the present calculation, the DFLUX user subroutine written in the Fortran language was used to implement the 3-D moving double ellipsoid heat source in the application of this 2-D finite element model: it was supposed that the heat source moves at some velocity and passed through the plane model. At this point, the double ellipsoid heat source intersected the 2-D model to form a cross-like section. Over time, the position of the heat source kept moving, the shape of the section also changed, and the internal heat distribution also kept changing, so as to realise the application of a 3-D moving body heat source in the 2-D model. This has been proved by many scholars [24,25,26], to not only ensure accuracy of calculated results but also improve the calculation efficiency.

**Figure 5:**
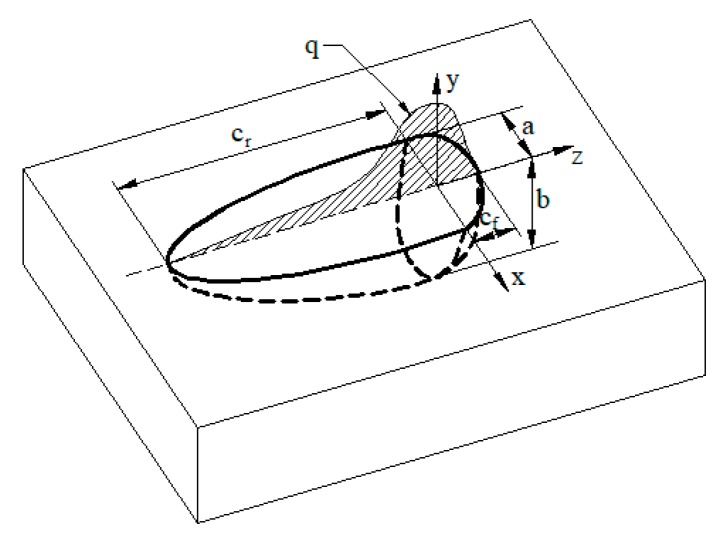
Heat source model used in the present study.

The double ellipsoidal heat source model, proposed by Goldak et al. (1984) [27], is shown in Figure 5. The rear portions and front of the heat source model are in two different ellipsoid quadrants. The power density distribution in the front portion is:

(1) $$q_{f}\left(x,y,z,t\right)=\frac{6\sqrt{3}f_{f}Q}{abc_{f}\pi \sqrt{\pi }}e^{-3x^{2}/a^{2}}e^{-3y^{2}/b^{2}}e^{-3{\left[z+v\left(\tau -t\right)\right]}^{2}/c_{f}{}^{2}}$$

The power density distribution in the rear portion is:

(2) $$q_{r}\left(x,y,z,t\right)=\frac{6\sqrt{3}f_{r}Q}{abc_{r}\pi \sqrt{\pi }}e^{-3x^{2}/a^{2}}e^{-3y^{2}/b^{2}}e^{-3{\left[z+v\left(\tau -t\right)\right]}^{2}/c_{r}{}^{2}}$$

where τ is a lag factor defining the position of the heat source at time *t* = 0; *f*_f_ and *f*_r_ are the forward and backward ellipsoidal energy fractions, *f*_f_ +*f*_r_ = 2; *a*, *b*, *c*_f_, and *c*_r_ are ellipsoidal shape parameters, which can have different values and are independent of each other. It should be noted that four octant ellipsoids may be used for heterogeneous welding, with each of *a*, *b*, and *c* taking independent values. Equations (1) and (2) have been applied in ABAQUS by coding the FORTRAN DFLUX user-subroutine [28]. The experimental determined information was used to calibrate the simulated heat source: the corresponding temperature cycles in the HAZ were correlated with the experimental measurements, with emphasis that was provided to cooling time and peak temperature.

**Figure 6:**
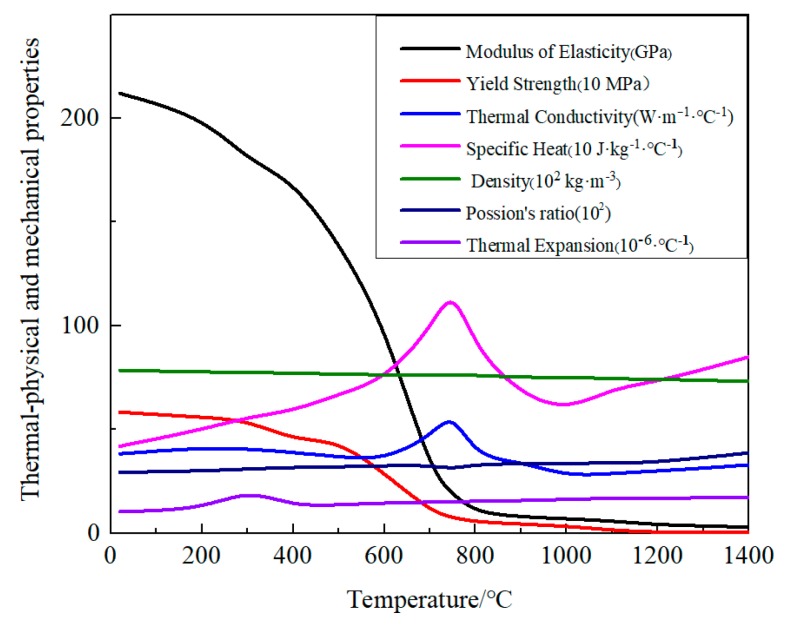
Thermo-physico-mechanical properties of SA738Gr.B.

The temperature-dependent and thermo-physico-mechanical properties of SA738Gr.B were used in this work, as shown in Figure 6 [29]. The welding material shall have the same material properties as the base material of SA738Gr.B.

### 3.3. Mechanical Analysis

To ensure the accuracy of solution, the thermal stress field of welding is considered as a transient problem of nonlinear material in this paper, and the elastic-plastic mechanical model is calculated by using incremental theory, and the following assumptions are made:

1. Welded parts under Von Mises yield criterion;
2. The behavior in the plastic zone of the welding part is subject to the plastic flow rule and isotropic hardening rule;
3. Elastic-plastic strain and thermal strain are inseparable;
4. Thermal properties and stresses/strains related to temperature change linearly in small time increments.

The temperature-dependent and thermal-physical mechanical properties of SA738Gr.B were used in this work (Figure 6) [29]. As there is little solid-state phase transformation in SA738GR.B, phase transformation data were not considered. The total strain can be divided into three components as given by Equation (3):

ε = ε^p^ + ε^e^ + ε^th^ (3)

where *ε*^th^, *ε*^e^, and *ε*^p^ are the plastic strain, thermal strain, and elastic strain, respectively. The modelling of elastic strain was performed based on the isotropic Hooke’s law with Poisson’s ratio and a temperature-dependent Young’s modulus. For the plastic strain, a plastic model was employed with temperature-dependent mechanical properties, an isotropic hardening model and Von Mises yield criterion. The thermal strain was calculated using the temperature-dependent coefficient of linear thermal expansion. Considering that the stresses S11, S22, and S33 were deduced in the 2-D model, the plane strain model could be used for simulation.

**Figure 7:**
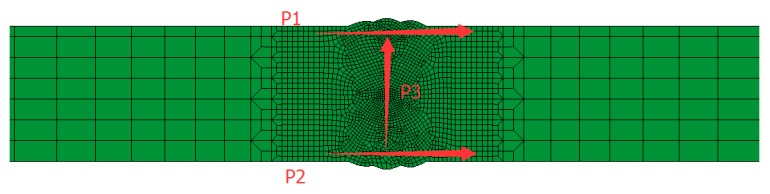
Schematic diagram of residual stress path selection.

Three paths were taken from the welding material section, namely, P1 on the upper surface, P2 on the lower surface, and P3 on the middle line of the welding seam (Figure 7). The values of three output paths of transverse stress S11, normal stress S22, and longitudinal stress S33 followed.

## 4. Results and Discussion

### 4.1. Results of Welding Temperature Field

The numerical simulation results and the accuracy of the double ellipsoidal heat source model were verified by comparing the simulated and experimental values of the temperature measurement points of manual welding.

According to the location of the temperature measurement points, the temperature measurement points in the numerical simulation of the welding temperature field were selected, and some peak temperature curves were taken and compared with the peak temperature curves of the actual welding temperature field (Table 3).

**Figure 8:**
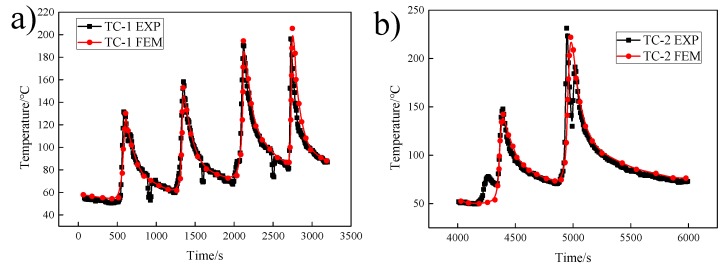

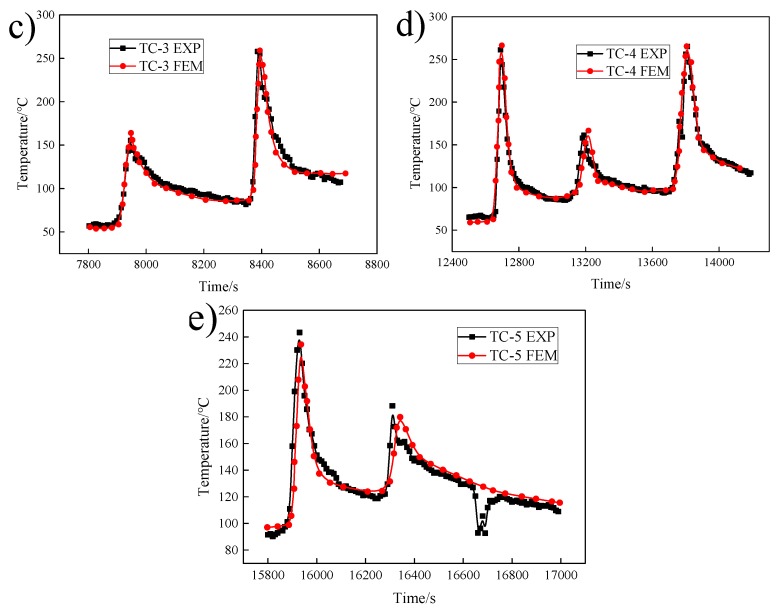
Comparison between the experimental value of temperature at TC-1 to 5 and numerical simulation: (**a**–**e**).

It can be seen from Figure 8 that the simulation results of the thermal cycle curve of the temperature measurement point were consistent with the test results, and errors within the allowable range may be caused by the failure of the manual welding process to maintain a constant speed, the interference of the arc in the welding, the time difference caused by replacing the welding rod, and the influence of factors such as the temperature change upon arc initiation and arc withdrawal.

**Figure 9:**
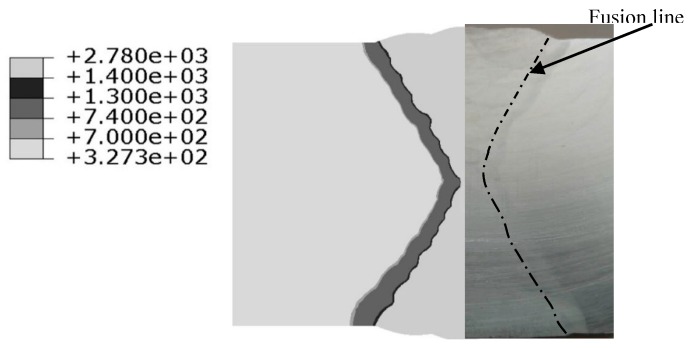
Comparison of fusion zone between EXP (experiment) and FEM (finite element modelling).

Figure 9 compares the fusion zones (peak temperature above 1400 °C) predicted by FEM and those measured by EXP. In this figure, the black dashed lines show fusion zone boundaries in the mock-ups, while the grey areas represent fusion zones as simulated by FEM. Through comparative analysis, it can be found that the FEM results are consistent with EXP data. The results show that fusion zones’ essential features are realised with existing calculation methods through the careful design of the type of heat source and the reasonable selection of the corresponding heat source parameters. It has been proved [30,31] that the thermal elasto-plastic behaviour at high temperatures (above the melting point) has very little influence on the deformation and final residual stress because both the yield strength and the elastic modulus are close to zero (Figure 6). It can be consequently inferred that the calculation accuracy of welding deformation and residual stress will not be significantly reduced when the shape and size of the welding zone (as calculated by FEA) are close to the shape and actual size of the welding zone [31]. As shown in Figure 9, the width of the heat affected zone predicted by FEM is around 3.0 to 3.2 mm, and the size of the HAZ was also similar to that found experimentally. In short, the model and size of the heat source used in manual welding simulation are reasonable.

### 4.2. Welding Stress Field Results

**Figure 10:**
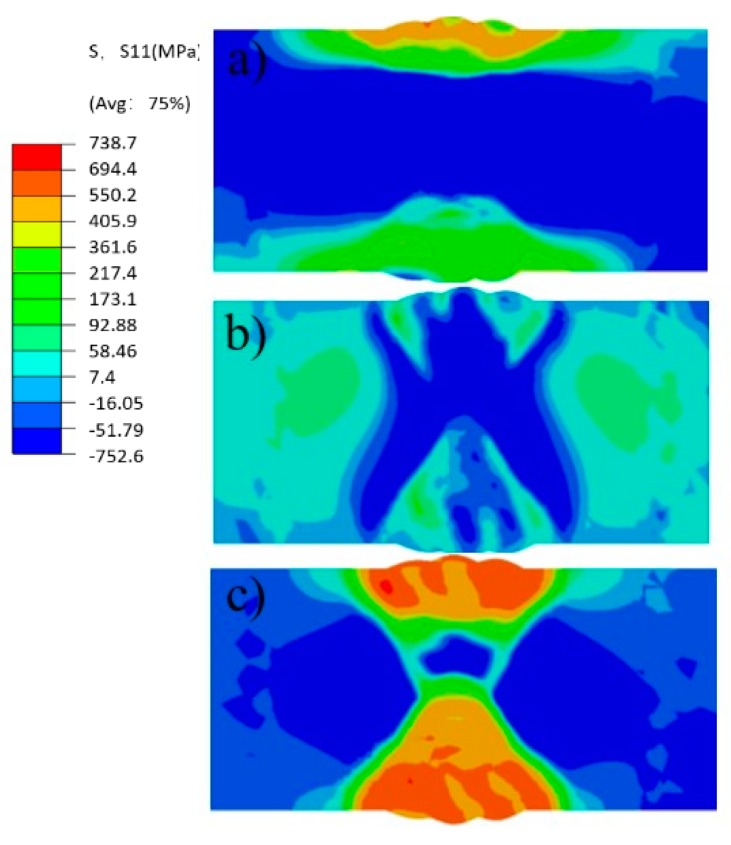
Stress contours (FEM) (**a**) S11; (**b**) S22; (**c**) S33.

Figure 10 shows the contours of weld residual stresses (as predicted by FEM): the transverse stress S11 and longitudinal stress S33 are larger than normal stress S22. The maximum value of transverse stress S11 is 584 MPa, and the maximum stress appears at the welding seam on the upper surface, while the stress at the welding seam on the lower surface is relatively low, and the compressive stress in the middle of the weld bead at the welding seam is about 600 MPa. The overall residual stress for normal stress S22 is low, and the peak value of the residual stress from welding is about 108 MPa, which appears at the edge of the welding groove on the butt-welding face, and the residual stress of the HAZ on both sides of the middle of the weld is low. The longitudinal stress S33 and its resultant welding residual stress are relatively high, and are distributed on both sides of the butt welding with the welding stress peak reaching about 717 MPa.

**Figure 11:**
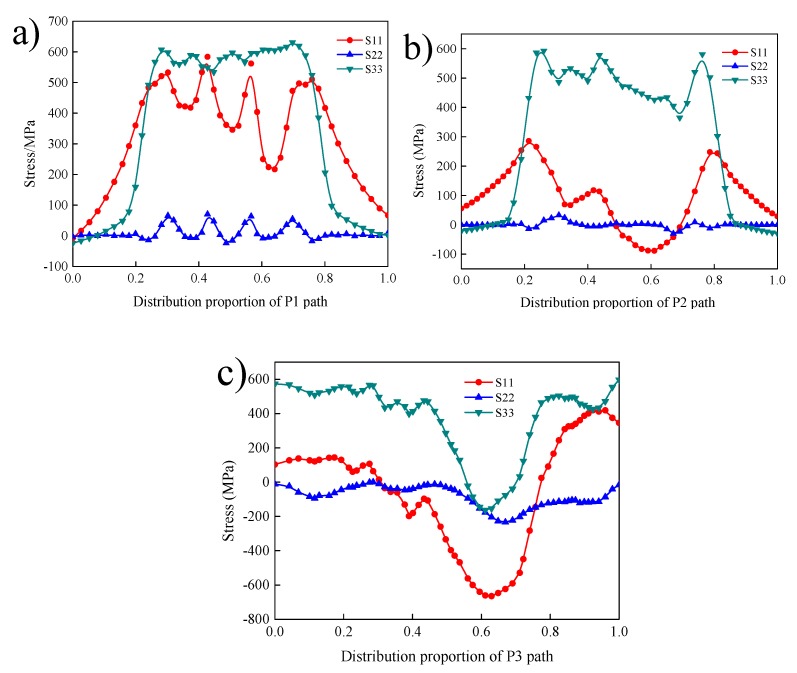
Residual stress along P1 (**a**), P2 (**b**), P3 (**c**) during a manual welding process.

Figure 11a shows the distribution of transverse stress S11, normal stress S22, and longitudinal stress S33 along P1: the normal stress S22 on path P1 is small, including both tensile stress and compressive stress, and all tensile stress and compressive stress are less than 100 MPa. The transverse stress and longitudinal stress at each point in the path of P1 are all tensile stress, and the peak value of the transverse stress S11 is in the HAZ, reaching about 629 MPa, while the peak value of the longitudinal stress S33 is in the weld zone, reaching 596 MPa.

Figure 11b shows the distribution of transverse stress S11, normal stress S22, and longitudinal stress S33 along P2: normal stress S22 is small, while the transverse stress S11 has both tensile stress and compressive stress components, and the longitudinal stress S33 is the largest, with the peak stress of about 600 MPa at the HAZ, and the tensile stress and compressive stress at other positions are all less than 600 MPa. The transverse force distribution at each point on P2 should be uneven, and the tensile stress appears in the welding HAZ, with a peak value of about 300 MPa.

Figure 11c shows the distribution of transverse stress S11, normal stress S22, and longitudinal stress S33 along P3. On P3, there was compressive stress in the middle of the weld and tensile stress at the edge of the weld. The peak compressive stress at the centre of the weld was about 700 MPa. The longitudinal stress S33 at each point on P3 has its minimum residual stress at the middle part of the weld, and the maximum residual stress at the outer edge of the weld is about 600 MPa. On P3, the peak value of lateral residual stress S11 appeared at the weld centreline, and the middle part of the weld was subjected to greater compressive stress. The longitudinal stress S33 on P3 is consistent with that on P1 and P2.

**Figure 12:**
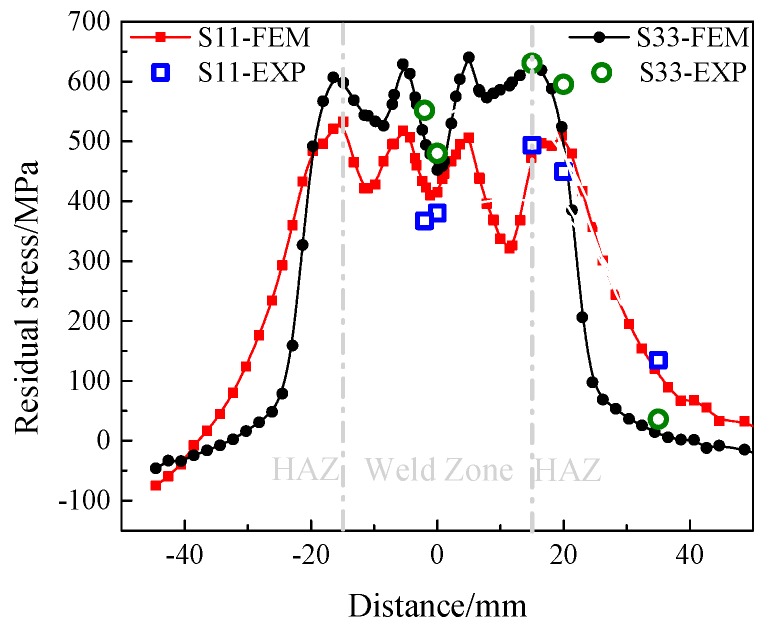
Comparison of residual stress between EXP and FEM along P1.

Figure 12 presents comparison of residual stress between EXP and FEM along P1. It can be seen from the figure that the measured values are similar to those from the numerical simulation analysis except for the transverse stress S11 at the two points of the weld seam. The measured values of transverse stress S11 at the welding seam are 367 MPa and 380 MPa respectively, while the simulated values are 413 MPa and 423 MPa, with differences of 12.5% and 11.3%, respectively. From the above analysis, the difference between experimental results and FEM output is within 12.5%: the present FE scheme is consequently proved to be appropriate for assessing the effects of welding sequence on residual stress.

### 4.3. Effect of Welding Sequence on Residual Stress

To study the influence of multilayer and multi-pass welding sequence on welding residual stress, different welding sequences were simulated. Welding sequence 1 is the welding sequence shown in Figure 1 and only one reverse pass was performed. After completing all passes (1 to 13) on one side, we welded the other side (passes 14 to 27) and gradually adjusted the frequency of flip welding, and set welding sequences 2 and 3, respectively, as shown below:

Welding sequence 1 (one-turn welding): 1~13,14~27

Welding sequence 2 (intermittent flip welding): 1~3,14~17,4~7,18~21,8~13,22~27

Welding sequence 3 (continuous flip welding): 1,14,2,15,3,16,4,17,5,18,6,19,7,20,

                     8,21,9,22,10,23,11,24,12,25,13,26,27.

**Figure 13:**
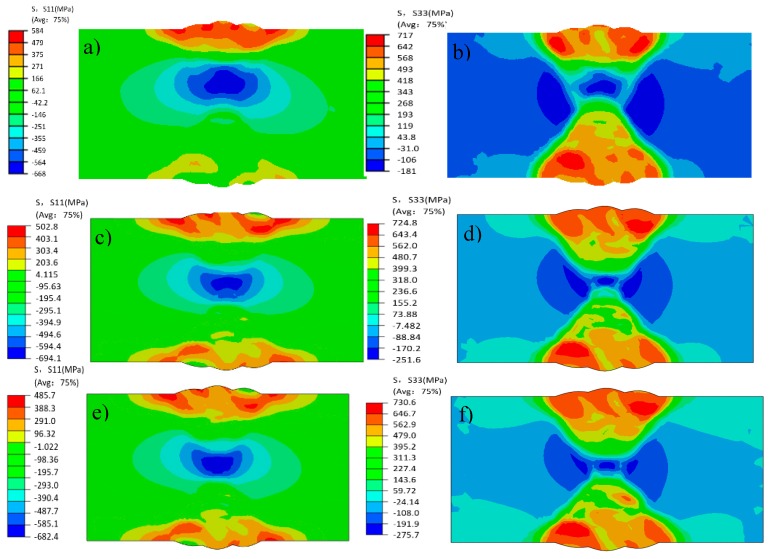
Contours of S11 and S33 stresses in different welding sequences: (**a**) S11 of Welding sequence 1, (**b**) S33 of Welding sequence 1, (**c**) S11 of Welding sequence 2, (**d**) S33 of Welding sequence 2, (**e**) S11 of Welding sequence 3, (**f**) S33 of Welding sequence 3.

From Figure 13 we can see that the sequence of welding passes affects the distribution of welding residual stress. Theoretically, by means of double-sided alternately symmetrical welding, the restraint of the weld bead is reduced by the base material, the cooling time of each pass is increased, and the welding residual stress is reduced. The actual results show that when the welding sequence changes from positive and negative alternating once to three times (that is, welding sequence 1 changes to WS 2), the peak transverse stress decreases from 584 MPa to 502 MPa, decreasing by 82 MPa (14%). When the welding sequence changed from positive and negative once to 13 times (that is, WS 1 changed three times), the peak transverse stress decreased from 584 MPa to 485 MPa, decreasing by 99 MPa (17%). Compared with the decrease in transverse stress, the peak value of longitudinal residual stress did not change significantly, but the binding force was reduced by adjusting the welding sequence, and the peak value of longitudinal stress was transferred from the toe of the weld and the area near the weld to the inside of the weld by deformation coordination, which greatly reduced the risk of surface stress corrosion and cracking. Therefore, in the formulation of the welding scheme, the use of double-sided alternating welding can effectively reduce the residual stress and deformation of the workpiece.

**Figure 14:**
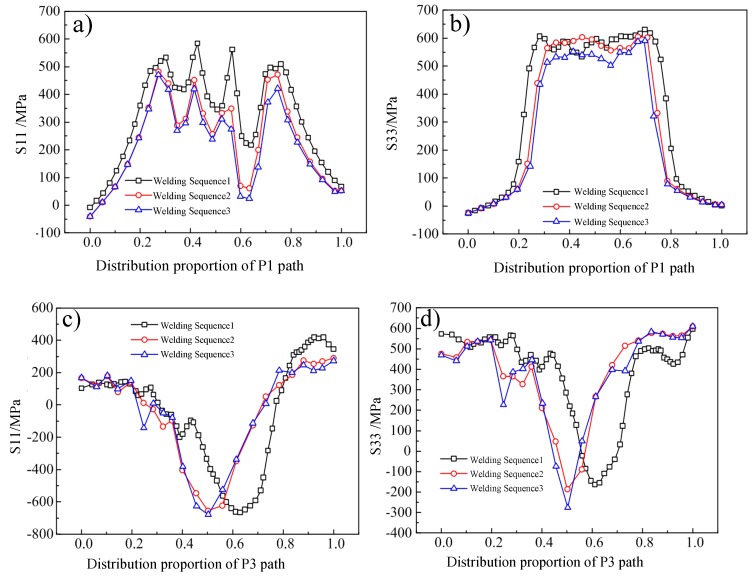
S11 and S33 stresses of different welding sequences along P1 and P3: (**a**) S11 along with P1, (**b**) S33 along P1, (**c**) S11 along P3, (**d**) S33 along P3.

Figure 14 shows S11 and S33 stresses in different welding sequences along P1 and P3. Based on different welding sequences in the curves of stress distribution in the thickness direction, comparison chart analysis can be found that compared with WS1, WSs 2 and 3 (i.e., increasing the number of welding passes alternately) can significantly reduce the residual stress peak value of longitudinal and transverse, but with the increase in number of symmetrical passes, from two to three, the welding sequence stress-reduction effect is weak or not significantly changed, so that can be used in the project. The effectively moderate number of alternations can be reached, but considering the point of view of engineering construction efficiency and construction difficulty, it is advisable to adopt symmetric alternation over three or four times (excessive alternating offers little benefit). In addition, when the number of alternations increases, such as from WS2 to WS3, the central axis of the residual stress distribution undergoes significant displacement, from 0.6 to 0.5 to the centre: this enhanced symmetry plays a role in stress coordination and redistribution.

## 5. Conclusions

The temperature field and stress field of the 2-D model was verified by experiment, and the residual stress fields of different welding sequences were studied. Based on the results obtained, the following conclusions can be drawn:

(1) The accuracy of the temperature field of the model was verified by comparing the thermal cycle curve with the heat affected zone.
(2) Through numerical simulation and analysis, it is found that the normal residual stress S22 changes little, which cannot be deduced from subsequent analysis. The transverse stress S11 is large on the surface of the weld and is largely tensile. The longitudinal stress S33 is larger at the centre of the weld and is largely compressive.
(3) The difference between FEM and experimental results (in terms of residual stress) is within 12.5%, demonstrating that the FE model is reliable.
(4) The sequence of welding passes directly affects the distribution of welding residual stress. From welding sequence 1 to welding sequence 3, transverse stress S11 changed significantly, decreasing by 14% and 17% respectively.
(5) In the welding process, the number of reversals should be increased as much as possible to reduce the welding residual stress.

**Figure 15:**
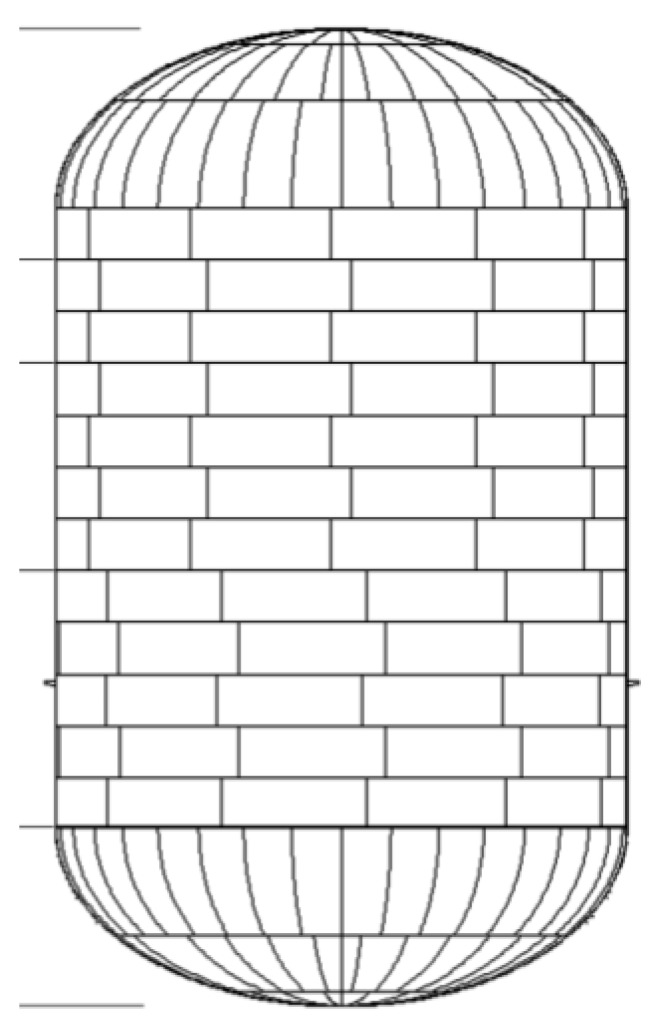
Steel containment structure (height, 73.6 m; diameter, 43 m).

It should be noted that the welding sequence was optimised following residual stress minimisation rules in the present study. In the actual manufacturing procedure, the selection of the welding sequence, nevertheless, still needs to consider actual working conditions, production efficiency, and other factors; however, as shown in Figure 15, in the actual manufacturing process, the selection of welding sequence still has to take into account the actual working conditions, production efficiency, and other factors.

## Figures and Tables

**Table 1:** Chemical composition of SA738GR.B and E9018-G-H4 (wt%).

	C	Si	Mn	P	S	Cr	Ni	Mo	V	Cu	Al	Nb
SA738GR.B	0.11	0.3	1.44	0007	0.002	0.18	0.51	0.2	0.04	0.02	0.028	0.02
E9018-G-H4	0.064	0.22	1.19	0.0084	0.0067	0.086	1.53	0.39	/	0.027	/·	/

**Table 2:** Welding parameters.

Weld Pass	Electric Current/A	Voltage/V	Welding Time/s	Weld Pass	Electric Current /A	Voltage/V	Welding Time/s
1	179	28.3	406	15	179	28.3	315
2	179	29.2	306	16	180	28.5	398
3	179	28.7	180	17	179	28.7	330
4	179	28.2	313	18	179	27.5	371
5	179	28.5	274	19	179	29.2	320
6	179	29.3	334	20	179	28.4	374
7	180	30.3	358	21	179	28.2	339
8	179	29.1	393	22	180	28.8	373
9	179	28.2	329	23	179	28.5	373
10	179	27.3	371	23	179	27.6	381
11	179	27.4	372	25	180	27.3	317
12	179	26.9	241	26	179	27.5	314
13	180	27.5	193	27	179	29.1	316
14	179	28.2	270				

**Table 3:** Temperature measurement points TC-1 to 5 corresponding to the number of weld passes and the peak temperature of thermal cycle.

	TC1				TC2		TC3		TC4			TC5	
Weld pass	1	2	3	4	8	9	10	11	16	17	18	20	21
EXP (°C)	145	163	186	193	150	239	170	258	262	157	252	242	187
FEM (°C)	143	160	183	197	147	234	173	261	267	163	257	235	178
